# Botulinum Toxin for the Treatment of Neuropathic Pain

**DOI:** 10.3390/toxins9090260

**Published:** 2017-08-24

**Authors:** JungHyun Park, Hue Jung Park

**Affiliations:** 1 Department of Anaesthesiology & Pain Medicine, Incheon St. Mary’s Hospital, College of Medicine, The Catholic University of Korea, Incheon 21431, Korea; happyjj@catholic.ac.kr; 2 Department of Anaesthesiology & Pain Medicine, Seoul St. Mary’s Hospital, College of Medicine, The Catholic University of Korea, Seoul 06591, Korea

**Keywords:** botulinum toxin, neuropathic pain, neuropathic pain treatment

## Abstract

Botulinum toxin (BoNT) has been used as a treatment for excessive muscle stiffness, spasticity, and dystonia. BoNT for approximately 40 years, and has recently been used to treat various types of neuropathic pain. The mechanism by which BoNT acts on neuropathic pain involves inhibiting the release of inflammatory mediators and peripheral neurotransmitters from sensory nerves. Recent journals have demonstrated that BoNT is effective for neuropathic pain, such as postherpetic neuralgia, trigeminal neuralgia, and peripheral neuralgia. The purpose of this review is to summarize the experimental and clinical evidence of the mechanism by which BoNT acts on various types of neuropathic pain and describe why BoNT can be applied as treatment. The PubMed database was searched from 1988 to May 2017. Recent studies have demonstrated that BoNT injections are effective treatments for post-herpetic neuralgia, diabetic neuropathy, trigeminal neuralgia, and intractable neuropathic pain, such as poststroke pain and spinal cord injury.

## 1. Introduction

Botulinum toxin (BoNT) has been used for decades in the treatment of diseases, such as dystonia or seizures, and cosmetic treatments. BoNT is useful in conditions such as strabismus because it causes long lasting but reversible paralysis via the administration of small amounts locally [1,2]. As BoNT purification technology develops, the range of use of this drug has been expanded, and the number of Food and Drug Administration (FDA)-approval diseases has also increased. Common to these applications is the fact that BoNT is absorbed from the neuromuscular junction or parasympathetic axon terminal to the motor neuron terminal because the toxin is responsible for the release of acetylcholine. It is important to note that these effects are not systematically redistributed but only localized. Numerous reports suggest that local administration of BoNT has a significant effect on neuropathic pain.

For a long time, the analgesic effect of Botulinum toxin A (BoNT-A) was considered to be due to the effect of muscle relaxation [3,4,5]. However, recent studies using BoNT in neuropathic pain models have demonstrated that BoNT has an analgesic effect independent of muscle relaxation by demonstrating dissociation of the duration of muscle relaxation and duration of pain relief [6].

In this paper, we investigate the mechanism of BoNT in neuropathic pain by examining the effects of the drug for intractable neuropathic pains, such as postherpetic neuralgia, diabetic neuropathy, complex regional pain syndrome, trigeminal neuralgia, phantom limb pain, spinal cord injury-induced neuropathic pain, and central poststroke pain.

## 2. Structure of Botulinum Toxin

BoNT is protein group produced by anaerobic bacteria called *Clostridium botulinum*, which has approximately 40 subtypes. However, seven serotypes are typically noted based on antigen specificity. Botulinum toxin A (BoNT-A) and B (BoNT-B) are the most commonly used drugs. Particularly, BoNT-A type has a molecular weight of approximately 900,000. BoNT-A is a double chain protein. The light chain (LC) is active, whereas the heavy chain (HC) is not active. BoNT binds to the acceptor at the nerve end and enters the nerve ending by receptor-mediated endocytosis. LC binds to the exogenous protein involved in exocytosis and breaks down the peptide bond of the protein transporter to block exocytosis and acetylcholine secretion. The C-terminal receptor-binding domain, which constitutes the heavy chain of BoNT, binds to ganglioside receptors and specific proteins on the cell membrane. This binding induces endocytosis of HC-LC. In general, acetylcholine binding to the acetylcholine receptor of the motor endplate is necessary for muscle contraction. At this time, the acetylcholine exocytosis process is necessary in presynaptic membrane. The normal acetylcholine exocytosis process requires three proteins: the synaptosomal associated protein-25 kDa (SNAP-25), syntaxin, and the vesicle-associated membrane protein (VAMP)/synaptobrevin in the presynaptic membrane. These proteins are called soluble N-ethylmaleimide (SNARE) proteins. As a zinc-dependent endoprotease, the LC of BoNT cleaves intracellular SNARE. This cleavage interferes with SNARE-mediated protein transport and transmitter release, blocking muscle innervation at the neuromuscular junction and resulting in flaccid paralysis [7,8]. This effect of BoNT LC is dependent on the serotype, but it persists from days to months [9,10].

BoNT-A and BoNT-B are effective in neuropathic pain. Mice can be treated with nerve ligation to induce mononeuropathy and cisplatin to induce polyneuropathy. BoNT-B improves allodynia and hyperalgesia [11]. A clinically reported case report demonstrates that BoNT-B improves pain and symptoms in complex regional pain syndrome (CRPS) patients with a lumbar sympathetic block [12].

## 3. Mechanism of Action of Botulinum Toxin for Neuropathic Pain (Experimental Study)

**Figure 1:**
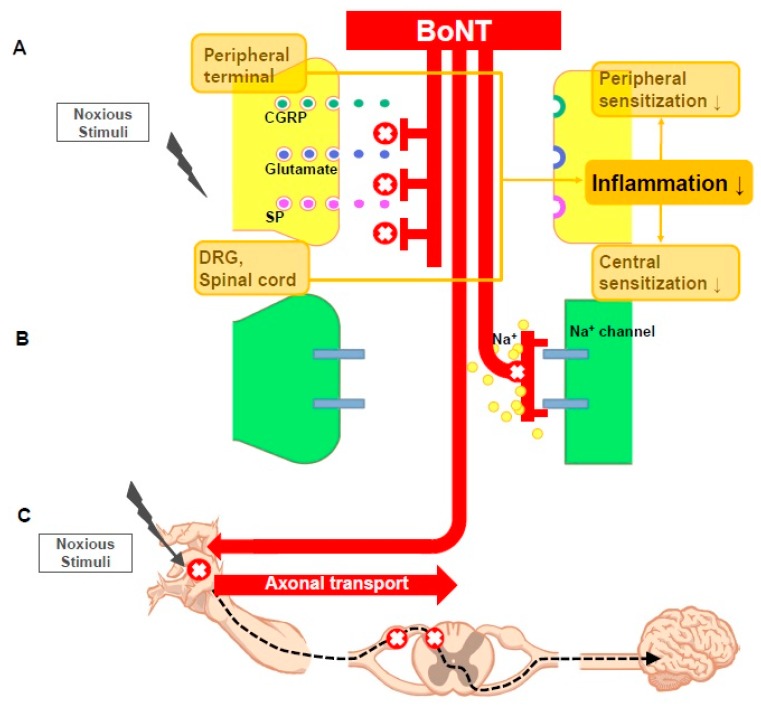
(**A**) Noxious stimuli cause inflammation through the release of neuropeptides and inflammatory mediators, which can cause peripheral sensitization. This action also occurs in DRG, dorsal horn of spinal cord and can lead to central sensitization. Botulinum toxin (BoNT) inhibits the release of pain mediators in peripheral nerve terminal, DRG, and spinal cord neuron, thereby reducing the inflammatory response and preventing the development of peripheral and central sensitization. Symbols; SP, substance P; CGRP, calcitonin gene related protein; DRG, dorsal root ganglion; (**B**) The hyperexcitability and spontaneous action potential mediated by the Na channel in peripheral sensory neuron contribute to the pathophysiology of neuropathic pain. BoNT can control neuropathic pain by blocking the Na channel; (**C**) Some of the BoNT appear to retrograde transport along the axons. SNAP-25 is cleaved in the dorsal horn of the spinal cord and central nuclei after a small amount of BoNT is administered to the periphery, thereby boosting the retrograde transport of BoNT.

BoNT also reduces and alters neuropathic pain in several animal models via the following mechanisms. BoNT inhibits the secretion of pain mediators (substance P, glutamate, and calcitonin gene related protein (CGRP)) from the nerve endings and dorsal root ganglions (DRG), reduces local inflammation around the nerve endings, deactivates the sodium channel, and exhibits axonal transport. We will review the various mechanisms by which BoNT reduces neuropathic pain (Figure 1).

### 3.1. BoNT Inhibits the Release of Pain Mediators from the Peripheral Nerve Terminal, DRG, and Spinal Cord Neuron

The effect of BoNT on the secretion of sensory neurotransmitters has been documented in several animal models. BoNT reduces normal CGRP release and capsaicin-induced DOA secretion and has additional effects on the TRPV1 pathway [13]. According to Meng et al., in a rat trigeminal ganglion sensory peptidergic neuron cell culture model, BoNT cleaves neuronal SNARE and blocks neurotransmitter secretion [14]. Durham et al. also reported a prophylactic advantage in migraine headaches via blocking the release of neuropeptides, such as CGRP from the trigeminal ganglion neuronal culture [15].

Fan et al. demonstrated that BoNT significantly reduces TRPV1 protein levels. Several studies demonstrated that TRPV1 plays a crucial role in arthritis pain, and this article examined the causal relationship between the antinociceptive effect of BoNT and the expression of TRPV1 in DRG of rats with arthritic pain. No significant changes in TRPV1 mRNA levels were observed via RT-PCR performed with different BoNT doses (1, 3, and 10 U); However, BoNT or TRPV1 protein levels were significantly decreased. This paper demonstrates the antinociceptive mechanism of BoNT by reducing TRPV1 expression by inhibiting plasma membrane trafficking after intra-articular administration [16].

### 3.2. BoNT Reduces Inflammation

Cyclophosphamide (CYP) was injected into the bladder of rats to induce CYP-induced cystitis, and HCL was injected into the bladder to induce acute injury. The bladder was harvested and compared with the Sham group. The cells were cultured in a solution containing BoNT to compare neurotransmitters. CGRP and substance P were significantly increased in the acute injury group compared with the control group, and substance P was significantly increased in the CYP-induced cystitis group. After exposure to BoNT, neurotransmitter levels were significantly reduced. In this article, we found that BoNT has an anti-inflammatory effect on chronic inflammation and acute injury [17]. In a chronic arthritis dog model, intraarticular BoNT injections are effective for up to 12 weeks [18,19]. The anti-inflammatory effect of BoNT reduces the release of peripheral neurotransmitters and inflammatory mediators. 

However, the effects are debated. Rojecky et al. injected carrageenan and capsaicin into the hindpaw of the rat, and rats were treated with BoNT five days before injection. No significant differences in edema and plasma protein extravasation were noted between the group injected with BoNT and the group without BoNT [20]. In addition, Sycha et al. reported that the BoNT group and the control group had no direct effect on acute, noninflammatory pain in the group treated with BoNT upon skin exposure to Ultraviolet B [21]. Chuang et al. measured cyclooxygenase-2 (COX-2) levels in the capsaicin-induced prostatitis model. COX-2 is a key enzyme that is an important mediator of inflammation and pain. COX-2 expression was induced as assessed by Western blotting or immunostaining. Inflammation was induced upon injection of capsaicin into the prostate of an adult male rat. Another group was pretreated with 20 U BoNT one week before injection of capsaicin. The expression of COX-2 was reduced in spinal sensory and motor neurons and the prostate in the pretreatment group [22].

BoNT also decreases local inflammation around the nerve terminal. According to the report of Cui et al., BoNT was administered to the footpads in formalin-inflammatory pain model rats. The antinociceptive effect started 5 h after BoNT treatment and persisted for greater than 12 days. In addition, edema was reduced, but no localized muscle weakness was observed. Formalin-induced glutamate release was also significantly reduced. This finding demonstrates that local inflammation around the nerve endings is reduced in the absence of obvious muscle weakness [23].

### 3.3. BoNT Deactivates Sodium Channels

BoNT also deactivates the sodium channel. Na current stimulates numerous cellular functions, such as transmission, secretion, contraction, and sensation. BoNT-A changes the Na current of a neuronal excitable membrane, which is different from that of local anesthetics, tetrodotoxin, and antiepileptic drugs that completely control the Na current via a concentration-dependent manner [24].

### 3.4. BoNT Exhibits Axonal Transport

BoNT exhibits axonal transport function from the periphery to the CNS, and administering BoNT to the facial and trigeminal nerve causes SNAP-25 cleavage in the central nuclei. In addition, a small amount of BoNT was injected into the hind limb, confirming the cleavage of the SNAP-25 in the ventral horn and the dorsal horn of the ipsilateral spinal cord, thereby demonstrating the retrograde axonal transport function of BoNT [25]. In addition, the BoNT effect on both sides has been reported after injecting BoNT on one side [26,27,28]. In animal studies, the anti-nociceptive effect of BoNT was studied in paclitaxel-induced peripheral neuropathy. The withdrawal nociceptive reflex was reduced after paclitaxel injection into the hind paw of the rat. BoNT was injected into one side, but the analgesic effect was observed on both sides. Diffusion into blood circulation may affect the central nervous system, but the dose was too low to cause systemic side effects. BoNT is also too large to pass the BBB, so the theory that BoNT is transmitted from the periphery to the central nervous system through the axon is possible [28]. To prevent retrograde axonal transport, Rojecky et al. confirmed the antinociceptive effect of unilateral transport of the axonic transport blocker colchicine in the ipsilateral sciatic nerve [26], which also demonstrated the retrograde axonal transport of BoNT. 

However, this notion is controversial. Tang et al. injected ^125^I-radiolabeled free BoNT into the gastrocnemius muscle of rats and rabbit eyelids and observed BoNT in various tissues at different time points. In both rabbits and rats, systemic effects were absent, and most of the toxins remained in the injection site. The authors concluded that most of the BoNT remained near the injection site and did not cause systemic toxicity [29]. 

Whether BoNT is transported retrograde from the injection site remains controversial. However, retrograde axonal transport has been demonstrated in numerous papers. Marinelli et al. analyzed the expression of cl-SNAP-25 from the nerve endings of the hind paw to the spinal cord after applying BoNT to the periphery. Immunostained cl-SNAP-25 was detected in all tissues. Additional experiments were performed to assess whether the growth state of Schwann cells interacts with BoNTs. As a result, BoNT regulated the proliferation of Schwann cells to inhibit acetylcholine release. This result demonstrates retrograde trafficking of BoNT [30].

## 4. Clinical Study of Botulinum Toxin for Neuropathic Pain

### 4.1. Trigeminal Neuralgia

A review of the efficacy of Botulinum toxin (BoNT) on trigeminal neuralgia (TN) has been reported in approximately 11 cases, including three RCT papers. This review includes the largest number of clinical trials for neuropathic pain for BoNT. In a randomized, double-blind, placebo-controlled study of 42 patients, Wu et al. performed a parallel design with intradermal or submucosal injection of 75 U of BoNT-A in 22 patients. Twenty patients in the control group received 1.5 mL saline. In the BoNT group, 68.8% of patients had a visual analog scale (VAS) reduction of greater than 50%. In the control group, a VAS reduction of greater than 50% was noted in 15% of the patients [31]. In addition, a randomized, double-blind, placebo-controlled study was performed in 84 adults with TN by Zhang et al. 28 control subjects were treated with saline, 27 with 25 U BoNT-A, and 29 with 75 U BoNT-A. The response rates in the 25 U and 75 U groups were 70.4% and 86.2%, respectively, which were significantly different from the control group (32.1%). However, no significant differences were noted between the two groups [32]. According to Zuniga et al., 20 patients received 50 U of BoNT-A, and 16 controls received the same dose of saline. VAS was 4.9 vs. 6.63 at two months follow-up. No significant differences were noted between the two groups. At three months, there was a significant difference at 4.75 vs. 6.94 [33].

Prospective, open, and case series for trigeminal neuralgia are reported in three studies. According to Bohluli et al., 15 TN patients were administered 50–100 U of BoNT-A in the trigger zone without any special injection mode. All patients reported a reduction in pain frequency and VAS score [34]. Zuniga et al. reported 12 trigeminal neuralgia patients who underwent subcutaneous injection in the trigger zone, and a reduction in VAS lasting greater than two months was noted in 10 patients [35]. Turk et al. also reported that injection of 50 U BoNT-A at 1.5–2 cm depth around the zygomatic arch was performed in eight patients, and the incidence of pain and VAS were reduced in all patients [36]. The above papers are summarized in Table 1. 

### 4.2. Postherpetic Neuralgia

Two BoNT RCTs for postherpetic neuralgia (PHN) have been reported. Xiao et al. performed a randomized, double-blind, placebo-controlled study of 60 patients with PHN. The following study groups were included: the BoNT group, 0.5% lidocaine group, and 0.9% saline group. These patients were treated 5 U/mL BoNT-A, 0.5% lidocaine and 0.9% saline in the affected dermatome, respectively. Follow-up was performed at one day, seven days, and three months after drug administration. The BoNT group exhibited significantly improved VAS and sleep quality compared with the other two groups [37]. In addition, Apalla et al. performed a randomized, double-blind, placebo-controlled study on 30 adults with PHN, and the affected sites were divided into a chessboard of 5 U BoNT-A per injection. Thirteen of the 15 patients had a VAS reduction of at least 50% lasting approximately 16 weeks and a significantly reduced the sleep score [38]. Previously, there were reports on the antinociceptic effect of BoNT. Liu et al. reported that the VAS decreased from 10 to 1 after BoNT-A injection into the lesion, and the effect persisted for 52 days [39]. Sotiriou et al. reported assessed a case series of three patients. The affected site was divided into a chessboard form using a total of 100 U BoNT-A with 5 U injected at each point. The VAS started to decrease in three days and continued to decrease for greater than two months [40]. These papers are summarized in Table 2.

### 4.3. Post-Surgical Neuralgia

Four reports on the efficacy of BoNT on post-surgical neuralgia, including RCT articles, have been published. RCT articles include post-herpetic neuralgia and post-traumatic neuralgia. According to Ranoux et al., 29 patients with focal painful neuropathy and mechanical allodynia were included in a randomized, double-blind, placebo-controlled study. Up to 20–190 U BoNT-A was injected into the pain site intradermally. The injections reduced VAS, burning sensation, and allodynic brush sensitivity and improved QOL [41]. Layeeque et al. also observed postoperative pain. In 48 breast cancer patients subject to mastectomy, 22 patients were treated with BoNT-A in the pectoralis major, serratus anterior, and rectus abdominis muscle, and 26 control group patients were not treated. The group treated with BoNT reported improved post-operative pain, and post-operative analgesic use was significantly reduced. In addition, the tissue expander was removed from one patient in the BoNT group and five patients in the control group. The BoNT group did not complain of any particular complications [42]. A case report described satisfactory results from subcutaneous injection of BoNT-A in a 67-year-old patient with post-thoracotomy pain for more than two years postoperatively. The pain site was divided into 1-square centimeter. Then, 2.5 U of BoNT-A was injected into the middle, and 100 U BoNT-A was administered in total. The patient reported improved pain after five days, and pain relief persisted for up to 12 weeks [43]. According to Rostami et al., eight cancer patients with persistent focal pain were treated with surgery or radiotherapy. BoNT-A was injected intramuscularly or subcutaneously into the localized pain area. All patients reported significant VAS improvement, and a significant improvement in QOL was also noted [44]. The above studies are described in Table 3.

### 4.4. Diabetic Neuropathy

Two randomized, double-blind, placebo-controlled studies used BoNT for pain control of diabetic neuropathy (DN). In a study of 20 DN patients, Yuan et al. reported that 4 U of BoNT-A per site (total 50 U) was administered to the dorsum of foot, and 44% of patients had a clear reduction in VAS lasting three months and improved sleep quality [45]. Ghasemi et al. conducted a study similar to the previous paper, except that the BoNT dose was 8–10 U per site in 40 DN patients. A decrease in neuropathic pain score (NPS) and Douleur Neuropathique 4 (DN4) scores were reported in that study [46]. A meta-analysis of these two articles concluded that DN has a significant association between BoNT and pain relief [47]. The above papers are described in Table 4.

### 4.5. Occipital Neuralgia 

Kapural et al. retrospectively analyzed six patients injected with 50 U BoNT-A in the occipital nerve and found that the VAS was significantly reduced. Five patients exhibited pain relief lasting greater than four weeks [48]. Taylor et al. reported that 100 U of BoNT-A was administered to the occipital protuberance in the prospective, open, and case series. Improvement in sharp/shooting pain was noted, but no definite improvement in dull/aching pain was indicated [49]. Occipital neuralgia has been assessed in only two case series without an RCT article, so these studies are insufficient to prove the effectiveness of BoNT. The above papers are also described in Table 5.

### 4.6. Carpal Tunnel Syndrome

Breuer et al. conducted a randomized, double-blind, placebo-controlled study of 20 patients. In this study, 2,500 U of BoNT-B or saline was injected into hypothena muscle and tentorium associated with carpal tunnel. Tingling sensation, pain, and pain related to improved sleep were noted, but there was no significant difference compared with the control group [50]. In a prospective, open, pilot study of five patients, a total of 30 U of BoNT-A was injected intracarpally. Of the five patients, three reported insignificant pain relief, and none had electrophysiological changes [51]. These results suggest that the use of BoNT in carpal tunnel syndrome is not effective. These papers are described in Table 6.

### 4.7. CRPS

Safarpour et al. reported that two patients with CRPS had a reduction of CRPS and myofascial pain with the intramuscular administration of 20 U BoNT-A per site and trigger point injection [52]. They also performed randomized, prospective, double-blind, placebo-controlled, open-label extension studies of BoNT in CRPS patients. Fourteen patients with CRPS were divided into the BoNT group (*n* = 8) and control group (*n* = 6). A total of 40–200 U (5 U per point) BoNT was administered to the affected area with allodynia. No difference was found between the interventional group and the placebo group, and this study was terminated early due to the intolerance of BoNT [53]. In another study, lumbar sympathetic block was performed in a randomized, double-blind, placebo-controlled crossover study. Patients received standard LSGB on one side, and 10 mL of 0.5% bupivacaine was used. The same patient was injected with a crossover (another side) injection of 75 U BoNT-A in 10 mL of 0.5% bupivacaine. The control group has a median of 10 days, whereas the BoNT group has a median of 71 days [54]. In a case series published by Choi et al., two patients who experienced short-term effects on the lumbar sympathetic block were injected with 5000 U of BoNT-B in 0.25% levobupivacaine with a lumbar sympathetic block. VAS, allodynia, edema, coldness, and analgesic drug usage were reduced [12]. In a prospective, open case series of 11 patients with CRPS symptoms in upper limb girdle muscles, a total of 300 U of BoNT-A was administered to the pain-related muscles at 25–50 U. All patients exhibited improved VAS, allodynia, hyperalgesia, and skin color after 6–12 weeks [55]. In a retrospective, uncontrolled, unblended study of 37 patients, as a result of administering a total of 100 U of BoNT-A (10–20 U per pain site), 97% of patients reported pain reduction, and the average pain score decreased by 43% [56]. Except for one negative study, positive results have been published. However, these studies include a low class papers, and the effect of BoNT in CRPS patients has not been proven. These papers are summarized in Table 7.

### 4.8. Phantom Limb Pain

In a prospective, randomized, double-blind pilot study, 14 patients with phantom limb pain were treated with 50 U per site for a total of 250–300 U BoNT-A. In addition, a lidocaine and depomedrol mixture was administered at the focal tender point. VAS was assessed monthly in patients before and six months after treatment. Both groups reported improved pain. The BoNT group had an advantage over pain control during the 3–6 months, but phantom limb pain was not completely alleviated [57]. There is a case report in which the effect of BoNT was effective in reducing phantom limb pain for greater than 12 months. In total, 25 U of BoNT-A was injected into the trigger point of the stump at four sites, and the patient was able to reduce the pain medication given that the pain was significantly eliminated [58]. The effect of BoNT on phantom limb pain cannot be verified because only low-grade studies on phantom limb pain have been reported. The above papers are also listed in Table 8.

### 4.9. Spinal Cord Injury-Induced Neuropathic Pain

In a study of 40 patients with spinal cord injury-induced neuropathic pain, a randomized, double-blind, placebo-controlled design was used. In the BoNT group, 200 U BoNT-A was divided into 40 sites, and 4 mL of saline was administered to the control group in a similar manner. Pain intensities were assessed using VAS, the Korean version of the short-form McGill Pain Questionnaire (SF-MPQ), and the Korean version of the World Health Organization Quality of Life (WHOQOL-BREF) questionnaire. The same procedure was performed at baseline and four and eight weeks. The BoNT group exhibited a statistically significant decrease in VAS at four and eight weeks compared with the placebo group, and SF-MPQ was also significantly reduced compared with the placebo group. However, there was no significant difference between the control group and the BoNT group in the Korean version of the WHOQOL-BREF, which assesses physical health, psychological social relationship, and environmental domains [59]. A similar paper was published in 2017, and a randomized, double-blind, placebo-controlled study was performed in 44 patients with spinal cord injury-induced neuropathic pain. The BoNT group received 200 U of BoNT-A at the pain site, and the control group received the same amount of saline at the pain site. Unlike the above paper, patients received the same treatment once daily for eight weeks. The primary outcome of pain was measured on a VAS scale, and the secondary outcome was measured by the SF-MPQ and the WHOQOL-BREF questionnaire. At four and eight weeks, both primary and secondary outcomes were measured and evaluated. No adverse effect was noted in both groups. VAS and SF-MPQ were significantly decreased in the BoNT group compared with placebo group at four and eight weeks, respectively. The difference from the above paper is that the WHOQOL-BREF also exhibited a statistically significant decrease compared with the placebo group [60].

In addition, there have been several case reports of neuropathic pain associated with spinal cord injury. Jabbari et al. reported that two patients who had burning pain and allodynia after spinal cord injury injected with 5 U of BoNT-A at 16–20 sites in the pain site maintained significant VAS reduction for greater than three months [61]. Han et al. mentioned that 20 U of BoNT-A was injected into 10 painful areas in patients with spinal cord injuries, and VAS was decreased from 96 to 23 [62]. The use of BoNT for spinal cord injury is considered to be effective based on a statistically significant RCT journal report. These papers are listed in Table 9.

### 4.10. Central Poststroke Pain

Poststroke patients often use BoNT due to poststroke spasticity. However, some recent reports have reported that BoNT is used for central poststroke pain control. Shippen et al. injected BoNT in patients with elbow flexor spasticity with central poststroke pain. The patients had severe neuropathic pain at the site of the spasticity and received 100 U BoNT-A of Biceps Brachii, 75 U Brachialis and 25 U Brachioradialis. After the second day, the pain was reduced, and the spasticity was improved one week after administration. The patients repeat BoNT every three months to control pain [63]. Barbosa et al. also published a case report in which an analgesic effect was obtained using BoNT-A in patients with central poststroke pain. In two patients with stroke, injection of BoNT-A 200 U into the affected area under EMG guidance resulted in a decrease in NRS after a 3-month follow-up [64]. A randomized, double-blind, placebo-controlled trial of 273 patients with poststroke spasticity was performed. In total, 74.3% of the patients had stroke-related pain, and 47.3% were suffering from greater than NRS 4. Patients were divided into two groups: BoNT-A and standard care vs. placebo and standard care. The degree of pain was compared 12 weeks from the baseline, and the BoNT group reported significantly less pain compared with the placebo group. The reduction in pain persisted for up to 52 weeks [65]. This is the first RCT assessing the control of neuropathic pain with BoNT in patients with poststroke spasticity. Therefore, BoNT may be effective in patients with central poststroke pain. The above papers are summarized in Table 10.

## 5. Adverse Effects

BoNT-A has minimal irreversible medical adverse effect. Regarding the use of BoNT in cervical dystonia, side effects, including neck muscle weakness, dysphagia, pain during swallowing, and flu-like symptoms, are rarely reported. The use of BoNT in blepharospasm and cerebral palsy is associated with unilateral or bilateral ptosis, hematoma, and lower limb weakness and pain. When BoNT is used in neuropathic pain, relatively minor complications, such as antibody formation and immune-related complications, are reported when a small amount of BoNT-A enters the circulatory system [66]. BoNT-B can also be used to obtain effective results when neutralizing antibodies are present in BoNT-A, and the effect is reduced. [67,68].

## 6. Conclusions

Before beginning BoNT therapy, patients with neuropathic pain require a careful assessment of functional limitations, goals, and expected outcomes. The guidelines of the American Academy of Neurology recommend the use of BoNT-A in neuropathic pain as follows. In postherpetic neuralgia, trigeminal neuralgia, and spinal cord injury-induced neuropathic pain, BoNT is effective (Level A) and BoNT is probably effective in post-surgical neuralgia, diabetic neuropathy, and central poststroke pain (Level B). In neuropathic pain, such as occipital neuralgia, CRPS, and phantom limb pain, a large and well-designed blinded and randomized controlled trial is needed to evaluate the effect of BoNT. The route of administration of BoNT is different for each article. There are no clinical guidelines for administration of BoNT for neuropathic pain. Most treatments are subcutaneous or intradermal, and BoNT is also injected intramuscularly or into the surrounding tissues. In some papers, BoNT is injected into the skin as a chessboard. In other studies, BoNT is directly injected into the nerve. In particular, the development of ultrasound technology can accurately inject drugs near the nerve, and BoNT injection near the nerve is emerging as an alternative method [69].

There is a need for comparative studies on whether these methods are effective and safe or which methods are more effective than others. In addition, studies should be carried out to compare the minimum doses that are effective. Large, well-designed clinical trials are needed to address these problems.

## Figures and Tables

**Table 1:** Botulinum toxin for trigeminal neuralgia.

Study Design	Number of Patients	Method of Injection (Total Volume)	Result	Reference
Randomized double-blind, placebo-controlled	42	Intradermal, submucosal (75 U/saline 1.5 mL)	50% VAS reduction 68.8% (Botulinum toxin (BoNT) group) 15% (Control)	[31]
Randomized, double-blind, placebo-controlled	84 (27 BoNT 25 U, 29 BoNT 75 U, 28 control)	Intradermal, submucosal (25 U/75 U/saline 1 mL)	Visual analog scale (VAS) reduction 70.4% (25 U) vs. 86.2% (75 U) vs. 32.1% (Control)	[32]
Randomized, double-blind, placebo-controlled	36 (20 BoNT, 16 control)	Intramuscular (50 U/saline 1 mL)	VAS (BoNT vs. Control) 4.9 vs. 6.63 (2 months) 4.75 vs. 6.94 (3 months)	[33]
Prospective, open, case series	15	Injected at the trigger zones (50–100 U)	All patients improved frequency and severity of pain attacks	[34]
Prospective, open, case series	12	Subcutaneous (20–50 U)	VAS reduced lasting more than 2 months in 10 patients.	[35]
Prospective, open, case series	8	Around zygomatic arch, 1.5–2 cm depth (50 U per point, total 100 U)	Incidence of pain and VAS were reduced in all patients.	[36]

**Table 2:** Botulinum toxin for postherpetic neuralgia.

Study Design	Number of Patients	Method of Injection (Total Volume)	Result	Reference
Randomized, double-blind, placebo-controlled	60	Subcutaneous BoNT 5 U, 0.5% lidocaine, 0.9% saline per site	Significantly VAS pain score was decreased and sleep time improved	[37]
Randomized, double-blind, placebo-controlled	30	Divided into chessboard 5 U per site	50% VAS reduction of 13 patients	[38]
Case report	1	Fan pattern injection 100 U	VAS decrease from 10 to 1 Lasted for 52 days	[39]
Case series	3	Divided into chessboard 5 U per site (100 U)	VAS decrease and continued to 2 months	[40]

**Table 3:** Botulinum toxin for post-surgical neuralgia.

Study Design	Number of Patients	Method of Injection (Total Volume)	Result	Reference
Randomized, double-blind, placebo-controlled	29 (4 Postherpetic neuralgia, 25 Post-traumatic, post-surgical neuropathy)	Intradermal (20–190 U)	Decrease VAS, neuropathic nature pain and improve in quality of life	[41]
Prospective, non-randomized, placebo-controlled	48 (22 BoNT, 26 control)	Intramuscular (100 U)	Post-operative pain and analgesic use was reduced	[42]
Case report	1	Subcutaneous Affected zone was drawn with divisions of approximately 1 cm^2^, 2.5 U per site (100 U)	Improvement in pain was about 50% as measured on the VAS and persisted at 12 weeks	[43]
Pilot, prospective	8	Intramuscular, subcutaneous (100 U)	All patients had VAS improvement	[44]

**Table 4:** Botulinum toxin for diabetic neuropathy.

Study Design	No. of Patients	Method of Injection (Total Volume)	Result	Reference
Randomized, double-blind, placebo-controlled, cross-over trial	20	Intradermal 4 U per site at dorsum of foot (50 U per each foot)	44.4% of the BoNT group experienced a reduction of VAS within 3 months.	[45]
Randomized, double-blind, placebo-controlled	40	Intradermal, dorsum of the foot, in a grid distribution pattern, total 12 sites 8–10 U per site	Decrease in neuropathic pain score and Douleur Neuropathique 4	[46]

**Table 5:** Botulinum toxin for occipital neuralgia.

Study Design	No. of Patients	Method of Injection (Total Volume)	Result	Reference
Case series	6	Occipital nerve block 50 U for each block (100 U)	Significant VAS reduction and pain relief lasting >4 weeks	[48]
Prospective, open, case series	6	Greater and lesser occipital nerve block (100 U)	Improvement in sharp/shooting pain, no definite improvement in dull/aching pain	[49]

**Table 6:** Botulinum toxin for carpal tunnel syndrome.

Study Design	No. of Patients	Method of Injection (Total Volume)	Result	Reference
Randomized, double-blind, placebo-controlled	20	Intramuscular, hypothena muscle, tentorium (2500 U)	No significant difference compared to the control group	[50]
Prospective, open, pilot	5	Intracapal 30 U for each carpal tunnel (60 U)	Three patients insignificant reduced pain, none had electrophysiological change.	[51]

**Table 7:** Botulinum toxin for complex regional pain syndrome (CRPS).

Study Design	Number of Patients	Method of Injection (Total Volume)	Result	Reference
Case series	2	Intramuscular Trigger point 20 U per site	Reduction of CRPS pain and myofascial pain	[52]
Randomized, prospective, double-blind, placebo-controlled, and open-label extension	14 (8 BoNT group, 6 control group)	Intradermal, subcutaneous Allodynia area 5 U per site (40–200 U)	No difference between BoNT group and placebo group, terminated study early.	[53]
Randomized, double-blind, placebo-controlled crossover	9 (18 cases)	Lumbar sympathetic block 75 U BoNT + 0.5% bupivacaine/0.5% bupivacaine	Longer duration of pain reduction (BoNT vs. control/71 days vs. 10 days)	[54]
Case series	2	Lumbar sympathetic block 5000 U BoNT-B + 0.25% levobupivacaine	VAS and CRPS symptoms were reduced.	[12]
Prospective, open case series	11	Affected site, 25–50 U per site (300 U)	All patients had improved VAS, allodynia, hyperalgesia, and skin color after 6 to 12 weeks	[55]
Retrospective, uncontrolled, unblended	37	Affected site, 10–20 U per site (100 U)	The 97% patients reduced pain. (average pain reduction of 43%)	[56]

**Table 8:** Botulinum toxin for phantom limb pain.

Study Design	No. of Patients	Method of Injection (Total Volume)	Result	Reference
Prospective, randomized, double-blind, pilot	14	Intramuscular/cutaneous/subcutaneous/neuroma (EMG guidance) 50 U per site (250–300 U)	Both groups improved pain and BoNT group had an advantage over pain control during 3–6 months but could not completely change phantom limb pain.	[57]
Case series	3	EMG guidance into points with strong fasciculation (500 U)	Phantom pain, pain medication could be reduced, the gait became more stable and the artificial limb was better tolerated.	[58]

**Table 9:** Botulinum toxin for spinal cord injury-induced neuropathic pain.

Study Design	Number of Patients	Method of Injection (Total Volume)	Result	Reference
Randomized, double-blind, placebo-controlled	40	Subcutaneous (200 U)	Significantly VAS was decreased at 4 and 8 weeks.	[59]
Randomized, double-blind, placebo-controlled	44	Subcutaneous (200 U) Once daily for 8 weeks	Significantly VAS was decreased at 4 and 8 weeks.	[60]
Case	2	Subcutaneous 5 U of BoNT at 16–20 sites	Significant VAS reduction for more than 3 months	[61]
Case	1	Subcutaneous 20 U of BoNT at 10 sites	VAS decreased from 96 to 23.	[62]

**Table 10:** Botulinum toxin for central poststroke pain.

Study Design	Number of Patients	Method of Injection (Total Volume)	Result	Reference
Case	1	Intramuscular Biceps Brachii 100 U, Brachialis 75 U and Brachioradialis 25 U	Pain was reduced after 2 days, spasticity was improved after 1 week.	[63]
Case	2	Intramuscular Affected muscle (200 U)	NRS reduction for more than 3 months	[64]
Randomized, double-blind, placebo-controlled	273 (139 BoNT, 134 control)	Intramuscular Dosing was determined by investigator, second injection was performed with an open label and at least 12 weeks after the first injection	Significantly VAS was decreased at 12 weeks and reductions in pain were sustained through Week 52.	[65]
